# Drying-Induced Changes in Metabolite Profiles and Antioxidant Activity of *Cordyceps militaris*: Insights from Integrated Metabolomics and Network Pharmacology

**DOI:** 10.3390/foods15122061

**Published:** 2026-06-07

**Authors:** Xiaodan Wu, Weidi Fu, Wen Zhang, Hao Yu, Jianshuang Zhang

**Affiliations:** 1 School of Life Sciences, Guizhou Normal University, Guiyang 550025, China; 2 The State Key Laboratory of Southwest Karst Mountain Biodiversity Conservation of Forestry Administration, School of Life Sciences, Guizhou Normal University, Guiyang 550025, China

**Keywords:** *Cordyceps militaris*, drying methods, bioactive compounds, metabolomics, antioxidant activity

## Abstract

*Cordyceps militaris*, a medicinal and edible mushroom, is renowned for its bioactive constituents and health-promoting effects. This study investigated the effects of vacuum freeze drying (VF), vacuum drying (VD), oven drying (OV), and sun drying (SU) on the metabolite profiles and antioxidant activities of *C. militaris*. VF showed the highest levels of total phenolics, total carotenoids, cordycepin, and N^6^-(2-hydroxyethyl)-adenosine, whereas VD better preserved total flavonoids. VF- and VD-treated samples also exhibited stronger antioxidant capacities than those processed by OV and SU in 1,1-diphenyl-2-picrylhydrazyl radical (DPPH•), 2,2′-azino-bis (3-ethylbenzothiazoline-6-sulfonic acid) radical (ABTS^•+^), hydroxyl radical (•OH), and ferric reducing antioxidant power (FRAP) assays. Metabolomics analysis identified 193 significantly altered metabolites after drying treatments. VF, VD, and SU increased carbohydrates, vitamins, and phenolic acids, while leading to reductions in amino acids, nucleotides, and fatty acids. KEGG analysis revealed that drying significantly affected pathways related to purine and pyrimidine metabolism, amino acid biosynthesis, and phenylpropanoid biosynthesis. Network pharmacology further identified 8 key compounds potentially associated with antioxidant effects through interactions with 37 core targets. These findings highlight the importance of selecting appropriate drying methods to preserve the bioactive compounds and functional quality of *C. militaris*.

## 1. Introduction

*Cordyceps militaris*, a renowned entomopathogenic fungus, has been extensively used as health-promoting food and folk medicine in East Asian countries. It contained a wealth of bioactive components like cordycepin, carotenoids, polysaccharides, and other secondary metabolites, which may offered health benefits related to anti-aging and enhanced exercise performance [1]. Furthermore, *C. militaris* is used in clinical practices to treat lung and renal dysfunction, high blood sugar and lipid levels, respiratory problems, and fatigue [2]. Nowadays, *C. militaris* is becoming increasingly popular and important in daily consumption, serving not only as a raw material for diverse functional food products but also as a common ingredient in culinary applications. Nonetheless, fresh *C. militaris* is highly perishable due to its high moisture content, which renders it susceptible to rapid spoilage and quality degradation [3]. Therefore, appropriate postharvest processing is essential for preserving both the storage stability and functional quality of *C. militaris*.

Drying is an important approach for the long-term preservation of mushrooms, effectively inhibiting enzymatic reactions and microbial growth [4]. Nevertheless, drying is a complicated process that can lead to the breakdown of nutrients and metabolites, resulting in changes in the final product’s quality, including color, texture, and flavor [5]. Conventional drying methods, such as sun drying and oven drying, are widely used because of their low cost and operational simplicity [6]. Vacuum freeze drying is generally considered an effective method for preserving the appearance, aroma compounds, and antioxidant activity of *C. militari* [7]. Vacuum drying, which is commonly used for heat- and oxygen-sensitive food materials, may provide a practical balance between processing efficiency and metabolite retention [8]. These differences in processing characteristics may result in distinct effects on the metabolite composition and functional quality of *C. militaris*. To date, the majority of studies have concentrated on investigating how different drying methods impact the physicochemical properties, main bioactive components, and flavor compounds of *C. militaris* [3,9]. However, the impact of widely used drying techniques on the overall metabolic changes in *C. militaris* has not been fully explored.

As a valuable medicinal mushroom, *C. militaris* exhibits diverse biological activities that are important indicators of its product quality. Among these activities, antioxidant capacity has received considerable attention because of its important role in promoting human health [2]. Preventing oxidative stress-related disorders, including cancer, neurodegenerative diseases, and cardiovascular conditions, is closely related to this activity. Research has previously indicated that the drying techniques used can have a significant impact on the antioxidant properties of *C. militaris*. For example, vacuum freeze drying has been shown to better preserve the scavenging activity and reducing capacity of *C. militaris* compared with oven drying [7], while microwave drying was reported to enhance free radical scavenging activity and ferric reducing antioxidant power [9]. To date, previous studies have mainly focused on changes in physicochemical properties, selected bioactive compounds, flavor compounds, or antioxidant indices of *C. militaris* under different drying treatments [3,7,9]. However, the global metabolic changes induced by commonly used drying methods and its association with antioxidant-related quality remain insufficiently understood.

With the rapid development of analytical technologies, metabolomics has become an effective approach for systematically profiling food constituents and understanding their functional properties. Non-targeted metabolomics using ultra-performance liquid chromatography-mass spectrometry (UPLC-MS) provides an efficient strategy for the comprehensive profiling of metabolites in food resources [10]. It has also been employed to investigate the effects of drying on various mushroom and plant materials, revealing significant alterations in nutritional constituents and secondary metabolites [11]. However, metabolomics mainly describes chemical variation and does not directly explain the potential functional relevance of key metabolites. Network pharmacology can serve as a complementary predictive strategy for linking food-derived metabolites with potential biological targets and pathways associated with antioxidant and health-promoting effects [12]. Therefore, integrating metabolomics with network pharmacology may provide a more systematic framework for linking drying-induced metabolic changes with antioxidant-related quality changes in *C. militaris*.

Based on the above, this study aimed to investigate how different drying methods affect the metabolite profile of *C. militaris* and how these metabolic changes are associated with antioxidant-related quality. Specifically, the objectives were to: (1) investigate the impact of various drying processes on the metabolite profiles of *C. militaris*; (2) identify the differential metabolic compounds between different dried *C. militaris* and fresh samples; (3) predict the potential antioxidant-related targets and functional associations of key metabolites through network pharmacology analysis. The findings are expected to provide mechanistic insights into how drying processing influences the functional quality of *C. militaris*, thereby offering a scientific basis for optimizing drying strategies and preserving its bioactive components.

## 2. Materials and Methods

### 2.1. Materials

Fresh *C. militaris* fruit bodies, consistent in size and maturity, were sourced from: Guizhou Guiwang Biotechnology Co., Ltd. (Zunyi, China). Samples were rinsed twice with deionized water, drained and then divided into five groups, including fresh materials (FR), vacuum freeze drying (VF), oven drying (OV), sun drying (SU), and vacuum drying (VD). FR samples were chopped and ground into powder using liquid nitrogen. For VF, the samples underwent an initial freezing at −80 °C for 4 h, followed by a 24 h freeze at −40 °C using a freeze dryer with a vacuum pressure of 20 Pa (YTLG-10A, Yetuo, Shanghai, China). VD samples were dried in a vacuum drying oven (DZF-6012, YiHeng, Shanghai, China) at 40 °C under reduced pressure (135 Pa) for 20 h. The OV group was dried in a hot-wind circulation oven (BPG-9106B, YiHeng, Shanghai, China) at 50 °C, with drying time of 3–5 h. For SU, fresh samples were placed in a well-ventilated location with abundant sunlight for 3 days. The mean temperature recorded was 28 °C, and the relative humidity varied from 53% to 67%. The final moisture contents (wet basis) of VF, OV, SU, and VD samples were reduced to 7~8%, a standard that aligns with commercial *C. militaris* processing requirements. For comparability between fresh and dried samples, the moisture content of fresh *C. militaris* was assessed by drying in an oven at 105 °C until the weight remained constant, which showed a moisture content of 87.08% (wet basis). Accordingly, fresh samples were weight-adjusted to match the dry matter equivalent of dried samples for subsequent analyses. Subsequently, all dried *C. militaris* were pulverized into uniform fine powders with a high-speed grinder and sifted through an 80-mesh sieve. All samples in powdered form were preserved at −80 °C until further examination.

### 2.2. Chemicals and Reagents

Methanol of LC-MS grade was sourced from Dikma Technologies Inc. in Lake Forest, CA, USA, while acetonitrile was acquired from Fisher Scientific in Loughborough, UK. Methanol of HPLC grade was sourced from Sigma-Aldrich, located in St. Louis, MO, USA. The formic acid came from Tokyo Chemical Industry in Shanghai, China, while ultrapure water was generated using a Milli-Q system from Millipore, based in Bedford, MA, USA. Solarbio Science & Technology Co., Ltd. (Beijing, China) supplied gallic acid (purity over 98%), rutin (purity over 98%), Trolox (6-hydroxy-2,5,7,8-tetramethylchromane-2-carboxylic acid), and the Folin–Ciocalteu reagent. The compounds cordycepin (purity > 98.4%), N^6^-(2-hydroxyethyl)-adenosine (purity > 98%), and 1,1-diphenyl-2-picrylhydrazyl (DPPH) were sourced from Shanghai Yuanye Bio-Technology Co., Ltd. in Shanghai, China. Ferrozine, 2,2′-azino-bis (3-ethylbenzothiazoline-6-sulfonic acid) (ABTS), and potassium persulfate were acquired from Aladdin Biochemical Technology Co., Ltd. (Shanghai, China).

### 2.3. Determination of Total Phenolic, Total Flavonoid, and Total Carotenoid Contents

Preparation of the ethanolic extract was performed based on a previously described protocol with slight modifications [9]. Two hundred milligrams (dry weight) of each sample were blended in 10 mL of 70% ethanol-water (*v*/*v*) for one minute and then subjected to sonication at 55 °C for 30 min. After mixing, centrifugation was carried out at 4000 rpm for 10 min using a TD5 instrument (Bioridge, Shanghai, China). For further analysis of its composition, the collected supernatant was filtered using a 0.45 μm nylon membrane. The total carotenoids were extracted according to previous research [13]. Specifically, 1.6 g (dry weight) samples were completely homogenized with 100 mL of 73% ethanol and extracted using an ultrasonic bath at 54 °C with a power of 500 W for 30 min. After extraction, the sample was centrifuged at 4000 rpm for 10 min, and the supernatant was collected for further analysis. The Folin–Ciocalteu method, using gallic acid as a standard, was employed to measure the total phenolic content (TPC). An aluminum chloride colorimetric assay was employed to quantify the total flavonoid content (TFC), using rutin for calibration. The detailed procedures for determining TPC and TFC have been described in our previous work [10]. The total carotenoid content (TCC) in different *C. militaris* was determined spectrophotometrically based on the published method [13]. The experiments were performed in triplicate.

### 2.4. Determination of Cordycepin and N^6^-(2-hydroxyethyl)-adenosine Contents

Cordycepin and N^6^-(2-hydroxyethyl)-adenosine (HEA) contents were determined using a slightly modified method based on Wu et al. [3]. Briefly, 100 mg of each dried sample was mixed with 4 mL of double-distilled water and ultrasonically extracted at 60 kHz for 40 min. The extracts were then filtered through a 0.45 μm polyvinylidene fluoride membrane prior to analysis. All analyses were performed in triplicate. Quantitative analysis was conducted using an Agilent 1260 Infinity II LC system equipped with a quaternary pump and diode array detector (Agilent Technologies, Santa Clara, CA, USA). Chromatographic separation was achieved on a Hypersil BDS C18 column (250 mm × 4.6 mm, 5 μm) at 30 °C. The mobile phase consisted of 0.3% aqueous acetic acid (A) and methanol (B). A gradient elution program was used for separation: 0–10 min, 0–2% B; 10–20 min, 2–8% B; 20–30 min, 8–20% B; then holding at 20% B for 10 min. Operating at a flow rate of 1.0 mL/min, the system used an injection volume of 20 μL. A wavelength of 260 nm was used for the detection. Cordycepin and HEA were determined by external standard calibration.

### 2.5. Metabolite Extraction and Metabolomics Analysis

The extraction of metabolites from *C. militaris* was carried out according to our previously reported method with minor modifications [14]. Each sample, weighing precisely 50 mg (dry weight), was placed into a 2 mL centrifuge tube, followed by the addition of 1000 μL of 50% methanol. Vortexing was done for 30 s, and then ultrasonic extraction was conducted at 50 °C for 40 min. The samples were subsequently centrifuged at 12,000 rpm for 10 min under 4 °C. Afterward, 300 μL of the supernatant was passed through a 0.22 μm filter and examined with the UPLC-Q-Exactive-MS system. Each group included five biological replicates. Moreover, pooled quality control samples were created by mixing equal portions (20 µL) of supernatants from all samples, and these quality control (QC) samples were tested every five injections to ensure the stability of the analytical platform.

Metabolomics analysis was carried out using a UPLC Ultimate 3000 system coupled with an ACQUITY UPLC^®^ HSS T3 column (1.8 µm, 150 mm × 2.1 mm, Waters, Milford, MA, USA) and interfaced with a Thermo Q Exactive Focus mass spectrometer (Thermo Fisher Scientific, Waltham, MA, USA). For positive ion mode, the mobile phase consisted of solvent A (0.1% formic acid in water, *v*/*v*) and solvent B (0.1% formic acid in acetonitrile, *v*/*v*), whereas in negative ion mode, solvents C (water containing 5 mM ammonium formate) and D (acetonitrile) were used. In positive mode, the gradient program was structured as follows: from 0 to 1 min, 0 to 2% B; from 1 to 9 min, 2 to 50% B; from 9 to 12 min, 50 to 98% B; from 12 to 13.5 min, held at 98% B; from 13.5 to 14 min, reduced from 98% to 2% B; and from 14 to 20 min, 2% B. In negative ion mode, the gradient elution was set as follows: 0–1 min at 0–2% D, 1–9 min at 2–50% D, 9–12 min at 50–98% D, 12–13.5 min at 98% D, 13.5–14 min from 98% to 2% D, and 14–17 min at 2% D. The flow rate was set to 0.25 mL/min, and the injection volume was 2 μL, with the autosampler and column temperatures maintained at 8 °C and 40 °C, respectively. Electrospray ionization (ESI) was used for mass spectrometric detection, with capillary voltages of +3500 V for positive mode and −2500 V for negative mode. A temperature of 100 °C was maintained for the ion source, with the desolvation gas temperature at 350 °C. Data in full-scan mode were collected in both ionization modes over an m/z range of 81–1000 with a resolution of 70,000.

Raw MS data were first processed in R (version 4.3.2, https://www.r-project.org, accessed on 1 October 2024) using the XCMS (version 4.2.3) package for peak extraction, filtering, and alignment. Any features with a relative standard deviation exceeding 30% in QC samples were discarded. A data matrix with the mass-to-charge ratio (m/z), retention time (RT), and peak areas of the identified metabolites was then created for additional analysis. mass spectrometry (MS) and MS/MS spectral data were used for metabolite identification through comparison with a self-built BioDeep database and public resources such as Metlin (http://metlin.scripps.edu, accessed on 10 October 2024) and MassBank (https://massbank.eu/MassBank/, accessed on 10 October 2024). Mean-centering and unit variance scaling were applied to preprocess the data before conducting downstream analysis.

### 2.6. Measurement of Antioxidant Activities

The extracts used to evaluate antioxidant activity were prepared similarly to those for TPC and TFC. Antioxidant activity was evaluated using a series of in vitro methods reported in our previous study [10]. These assays encompassed tests for ferric reducing antioxidant power (FRAP), scavenging capabilities against 1,1-diphenyl-2-picrylhydrazyl radical (DPPH•), 2′-azino-bis (3-ethylbenzothiazoline-6-sulfonic acid) radical (ABTS^•+^), and hydroxyl radical (•OH), as well as the ferrous ion chelating activity (FICA). FRAP and free radical scavenging activities (DPPH•, ABTS^•+^, and •OH) were expressed in terms of Trolox equivalents per gram dry weight of *C. militaris* (mg TE/g). FICA values were expressed in terms of ethylenediaminetetraacetic acid (EDTA) equivalents per gram dry weight of the sample (mg EDTAE/g). Each assay was performed in triplicate.

### 2.7. Network Pharmacological Analysis

Antioxidant-related compounds’ canonical Simplified Molecular Input Line Entry System (SMILES) were collected from the PubChem database. The corresponding structural information was submitted to the SwissTargetPrediction and Traditional Chinese Medicine Systems Pharmacology (TCMSP) databases to predict potential molecular targets. A set of oxidative damage-associated genes was obtained from GeneCards and Online Mendelian Inheritance in Man (OMIM) using oxidative damage as the search term [15]. Following the removal of duplicates, Venn analysis was used to find the overlap between targets related to metabolites and those related to oxidative damage. Protein–protein interaction (PPI) analysis of the intersecting targets was conducted using the Search Tool for the Retrieval of Interacting Genes/Proteins (STRING) database. The clusterProfiler (version 4.19.7) package in R (version 4.5.2) was used to conduct GO functional enrichment and KEGG pathway analyses. The network illustrating interactions between metabolites and targets was finally developed and visualized with Cytoscape software (version 3.9.1).

### 2.8. Statistical Analysis

Multivariate analyses [Principal component analysis (PCA) and orthogonal partial least squares discriminant analysis (OPLS-DA)] were performed using ropls in R (version 4.3.2). Hierarchical clustering analysis (HCA) and K-means clustering were performed using stats (version 4.3.2) package in R (version 4.3.2). Statistical significance between the two groups was determined using Student’s test implemented in the stats (version 4.3.2) package of R (version 4.3.2, https://www.r-project.org/, accessed on 15 October 2024). Metabolites were considered significantly differential when false discovery rate (FDR) < 0.05 (Student’s test) and the variable importance in the projection (VIP) > 1.0. Analyses of Venn diagrams and heatmaps were carried out using the Hiplot online platform (https://hiplot.com.cn/, accessed on 20 October 2024). Pearson correlation analysis was conducted using the stats (version 4.3.2) package in R(version 4.3.2, https://www.r-project.org/, accessed on 27 October 2024). One-way analysis of variance (ANOVA) with Fisher’s least significant difference (LSD) post hoc test was used for statistical comparisons, utilizing SPSS version 24.0 (IBM Corporation, Chicago, IL, USA). Origin Lab’s software, version 2021, based in Northampton, MA, USA, was used to generate the bar charts.

## 3. Results and Discussion

### 3.1. The Changes of Total Phenolics, Total Flavonoids, Total Carotenoids, Cordycepin, and N^6^-(2-hydroxyethyl)-adenosine in C. militaris Under Different Drying Methods

**Figure 1:**
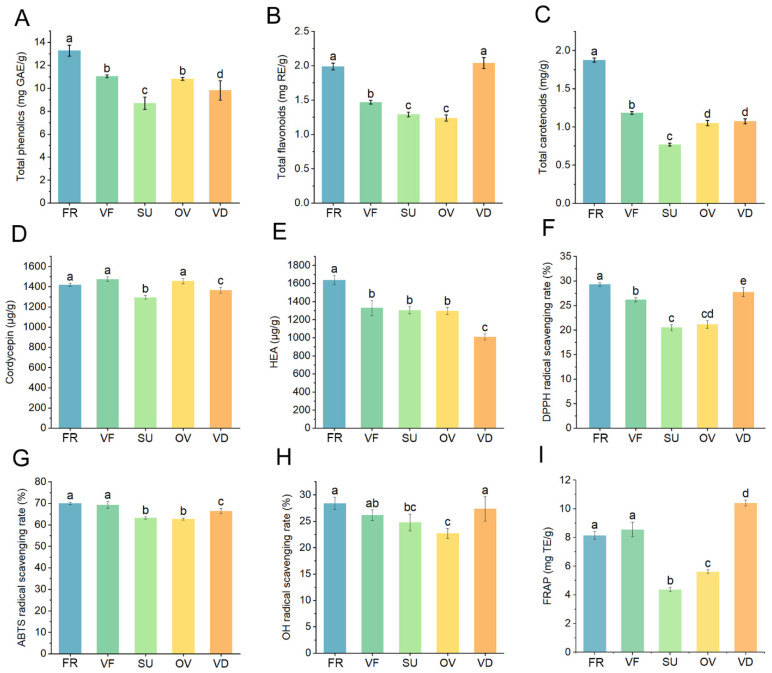
Effects of different drying methods on the bioactive compounds and antioxidant activities of *C. militaris*. (**A**) Total phenolic content. (**B**) Total flavonoid content. (**C**) Total carotenoids content. (**D**) Total cordycepin content. (**E**) Total N^6^-(2-hydroxyethyl)-adenosine content. (**F**) 1,1-diphenyl-2-picrylhydrazyl (DPPH) radical scavenging ability. (**G**) 2,2′-azino-bis (3-ethylbenzothiazoline-6-sulfonic acid) (ABTS) radical scavenging ability. (**H**) Hydroxyl radical scavenging ability. (**I**) Ferric reducing antioxidant power. Means with different superscript letters are significantly different (*p* < 0.05, ANOVA).

Thermal drying under high temperatures or extended drying durations generally leads to the degradation of phenolic compounds in mushrooms because of heat sensitivity, oxidative reactions, and enzyme activation. However, increased total phenolic content (TPC) has also been observed in oven-dried *Lentinula edodes*, potentially owing to the release of bound phenolics or the formation of new compounds during heating [16]. In this work, regardless of drying procedure applied, the TPC of different dried *C. militaris* was lower than the values of fresh materials (Figure 1A). This finding indicated that drying treatment consistently resulted in the loss of phenolics in *C. militaris*. In addition, freeze and oven drying led to the superior retention of TPC, followed by vacuum drying, and the lowest in sun drying (Figure 1A). Notably, the TPC of OV samples was comparable to those of VF group, suggesting a combined effect of both the degradation and elevation during thermal drying. For total flavonoid content (TFC), vacuum drying preserved similar levels to fresh samples, whereas freeze, sun, and oven drying significantly lowered it by 26.13%, 35.10%, and 37.71%, respectively (Figure 1B). Previous studies have shown that, because certain enzymes remain active at 40 °C, vacuum drying outperforms freeze-drying in preserving or enhancing certain classes of metabolites under certain conditions [17].

Carotenoid degradation frequently occurs during the postharvest handling of fruits, vegetables, and other plant materials. Figure 1C illustrated that the total carotenoid concentration in fresh and dried *C. militaris* spans from 0.77 to 1.88 mg/g of dry weight. As expected, total carotenoid content (TCC) was significantly reduced across all four drying methods when compared to fresh samples. The results corresponded with previous research demonstrating that carotenoids in *Boletus edulis* mushroom experienced significant losses after drying [18]. Moreover, variations in the effects of different drying methods on TCC were observed. Vacuum freeze drying retained the highest TCC, while sun drying resulted in the most significant reduction. Intermediate results were recorded for VD and OV. This trend aligns with findings from Li et al. [9], who concluded that vacuum freeze drying optimally preserves the TCC of *C. militaris*, followed by oven drying at 50 °C and sun drying. This could be explained by the high sensitivity of carotenoids to oxygen, heat, and light exposure, which readily alters their chemical structure and stability [19]. Consequently, the low-temperature and oxygen-restricted conditions during vacuum freeze drying help to mitigate such degradation, resulting in a comparatively high value of total carotenoids.

As shown in Figure 1D,E, the contents of cordycepin and HEA in different *C. militaris* were 1294.77~1503.24 μg/g and 1008.69~1638.39 μg/g, respectively. These results were slightly different from those of the previous report [20], which might be attributed to the fact that cordycepin and HEA accumulation in *Cordyceps* fungi were influenced by several factors, such as strains, cultivation conditions, and substrates. The effect of drying treatments on the content of cordycepin varied depending on the methods used. Vacuum freeze drying and oven drying had no significant impact on the level of cordycepin compared with fresh samples, while vacuum drying and sun drying caused significant loss. Consistent with previous findings [9], this study demonstrated vacuum freeze drying effectively maintained the amount of cordycepin. It was also observed that oven drying achieved a similar retention effect compared with freezing drying, which may be explained by the relative thermal stability of cordycepin during heat treatment [21]. Additionally, drying treatments resulted in a significant reduction in HEA content, irrespective of the method applied.

### 3.2. The Changes in Antioxidant Activities of C. militaris Under Different Drying Methods

It is widely acknowledged that the drying process will significantly influence the contents of antioxidant phytochemicals such as polyphenols and carotenoids in edible mushrooms [4]. The extracts of fresh *C. militaris* demonstrated the most potent antioxidant effects in DPPH, ABTS, and OH assays, with VF and VD samples trailing behind (Figure 1F–H). In contrast, the OV and SU samples demonstrated lower antioxidant activities across all assays (Figure 1F–I). These results indicated that vacuum freeze drying and vacuum drying may be more helpful in preserving the antioxidant components in *C. militaris* compared to other methods, which were consistent with previous findings that freezing dried *C. militaris* showed stronger DPPH• scavenging activity and reducing power than those of oven dried samples [9]. In addition, the drying treatment showed positive or negative effects on the FRAP of *C. militaris* depending on the methods used (Figure 1I). Vacuum drying significantly enhanced the reducing power, while sun drying and oven drying resulted in a marked reduction. Overall, the enhanced antioxidant activities observed in VF and VD samples may be attributed to the lower temperatures and oxygen-depleted conditions employed during drying processes, in contrast to oven drying or sun drying. These conditions effectively inhibit the degradation of heat-sensitive and oxygen-sensitive metabolites, thereby preserving their antioxidant properties [6]. Although vacuum freeze drying offered significant advantages in terms of active ingredient retention and product quality preservation, the high capital investment and energy consumption associated with the process remained major factors limiting its widespread adoption [22]. Therefore, vacuum drying, which ensured product quality and the stability of active ingredients while offering lower equipment and operating costs, was the drying technology with greater economic viability and potential for industrial application.

### 3.3. Global Metabolic Profiles of C. militaris

**Figure 2:**
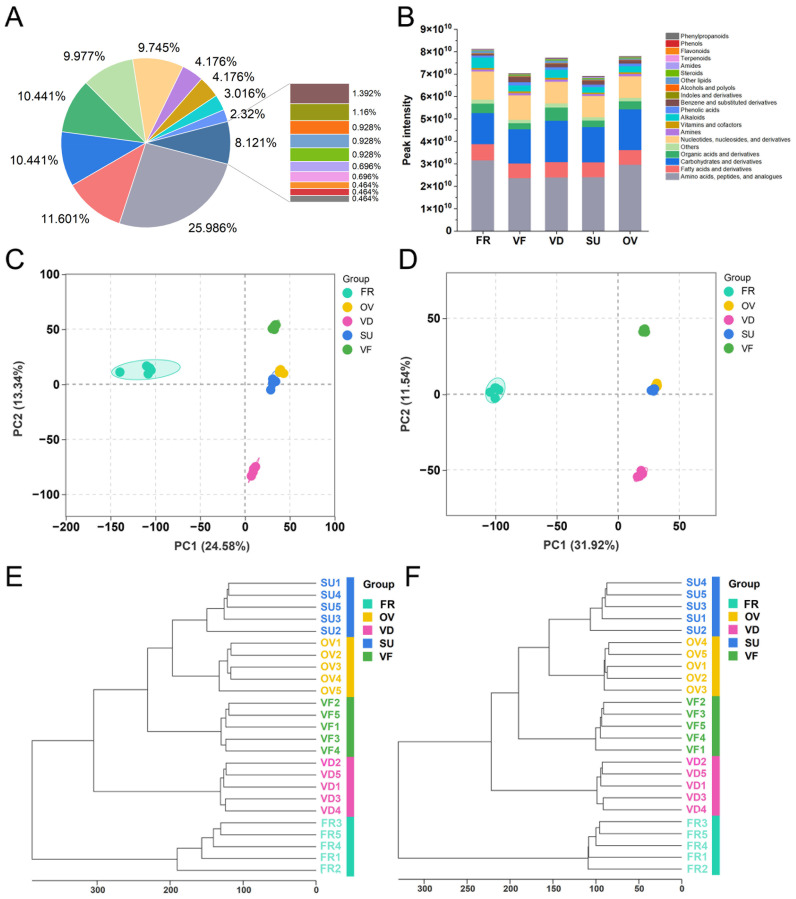
Metabolic profiles of *C. militaris* before and after drying. (**A**) Class distribution of the identified metabolites. (**B**) The relative abundance of each class of metabolites. Principal component analysis (PCA) score plots of *C. militaris* under different drying treatments in positive (**C**) and negative (**D**) ion modes. Results of hierarchical clustering analysis based on the identified metabolites in positive (**E**) and negative (**F**) ion modes.

Untargeted metabolomic profiling using UPLC–MS was performed to investigate the effects of drying on the metabolic profiles of *C. militaris*. UPLC–MS analysis detected 14,292 and 8300 ion features in positive and negative ion modes, respectively. All QC samples overlapped in the PCA score plot, revealing the stability of the instrument (Figure S1A,C). After normalization, 83.8% and 79.2% of peaks exhibited a relative standard deviation (RSD) below 30% in positive and negative modes, respectively, indicating good reproducibility of the metabolomics data (Figure S1B,D). By comparing the MS/MS spectra of samples to both public and self-constructed databases, 431 metabolites were tentatively identified and annotated (Table S1). These metabolites could be classified into several groups, predominantly including amino acids and derivatives, fatty acids and derivatives, carbohydrates and derivatives, organic acids and derivatives, and nucleotides and derivatives (Figure 2A). Comparative analysis revealed that the relative content composition of these metabolite classes remained consistent across fresh and dried samples (Figure 2B). Notably, fresh *C. militaris* displayed the highest total relative content of all identified metabolites, followed by VD, OV, and VF samples, whereas SU group showed the most significant reduction in metabolite levels.

### 3.4. Differentially Accumulated Metabolite Analysis of C. militaris After Drying

The drying process may trigger enzymatic and non-enzymatic reactions, resulting in pronounced changes in primary and secondary metabolite composition. To explore the similarity and differences among different *C. militaris*, PCA and HCA based on the features obtained from UPLC-MS were carried out. Figure 2C,D showed that all samples were separated into four groups according to their metabolite accumulation profiles. The samples of OV and SU clustered closer together, suggesting the high similarity in terms of their metabolic characteristics. Consistent with the PCA results, the fresh samples were clearly distinguished from the dried samples in HCA (Figure 2E,F). Furthermore, *C. militaris* samples processed by different drying methods were clustered separately, indicating that drying methods also obviously influenced the chemical profiles of *C. militaris*. Similar results have been reported in previous studies, in which the levels of flavor compounds in *C. militaris* altered obviously when processed by different drying methods [8].

OPLS-DA is a supervised multivariate statistical method that can effectively filter irrelevant variation in the dataset not related to the sample class. In this work, four pairwise comparisons between fresh *C. militaris* and different dried samples were performed using an OPLS-DA model. Figure S2A–H showed that the first principal component (PC1) of the OPLS-DA models clearly separated fresh and dried *C. militaris* samples. The *R^2^X* of all OPLS-DA models ranged from 0.522 to 0.538, reflecting that the models could explain a significant portion of the variance in the predictor variables. Both the *R^2^Y* and *Q^2^* values of the OPLS-DA models were greater than 0.955, demonstrating the good reliability and predictability of the models [10]. The reliability of the OPLS-DA models was further evaluated using 200-permutation tests. As shown in Figure S3A–H, the *Q^2^* regression lines exhibited negative intercepts, and all permuted *Q^2^* values were lower than those of the original models, indicating that the models were robust and not overfitted.

### 3.5. The Effect of Drying Treatment on the Metabolic Profiles of C. militaris

**Figure 3:**
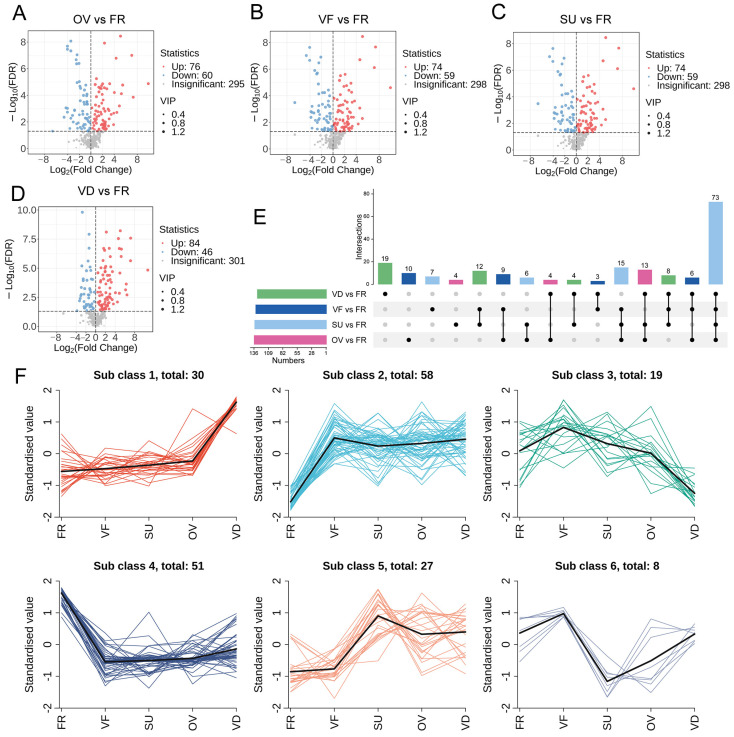
Identification of the significantly differential metabolites between fresh and dried *C. militaris*. Volcano plots of the differential metabolites in comparisonsoven drying (OV) vs. fresh materials (FR) (**A**), freeze drying (VF) vs. FR (**B**), sun drying (SU) vs. FR (**C**), and vacuum drying (VD) vs. FR (**D**). (**E**) The upset diagram of differential metabolites in the four comparison groups. (**F**) K-means clustering analysis of differential metabolites. Each colored line represents the trend of a metabolite during the drying process.

The significantly differential metabolites (SDMs) contributing to the variation between fresh and dried *C. militaris* were selected based on the variable importance in the projection (VIP) larger than 1.0 and FDR less than 0.05. There were 136, 133, 135, and 130 metabolites that exhibited differential accumulations in the OV vs. FR, VF vs. FR, SU vs. FR, and VD vs. FR comparisons, respectively (Figure 3A–D). A total of 193 SDMs were identified from the four comparisons, of which 73 metabolites were common across all four drying methods when compared to fresh samples (Figure 3E). In addition, there were 19, 7, 4, and 10 differential metabolites specific to each of VD vs. FR, VF vs. FR, SU vs. FR, and OV vs. FR, respectively (Figure 3E). The accumulated patterns of 193 SDMs were further explored using K-means clustering analysis, identifying six distinct subclasses. As illustrated in Figure 3F, 51 SDMs in subclass 4 showed higher contents in fresh *C. militaris* compared to those in dried samples. In contrast, the levels of SDMs in subclass 2 and subclass 5 increased in all dried samples. Notably, the SDMs in subclass 1 displayed distinct increasing trend in vacuum dried *C. militaris*. Overall, these SDMs are often associated with the nutraceutical properties or biological activities of *C. militaris*, primarily comprising amino acids, carbohydrates, fatty acids, nucleotides and nucleosides, vitamins, and phenolic acids (Table S2).

**Figure 4:**
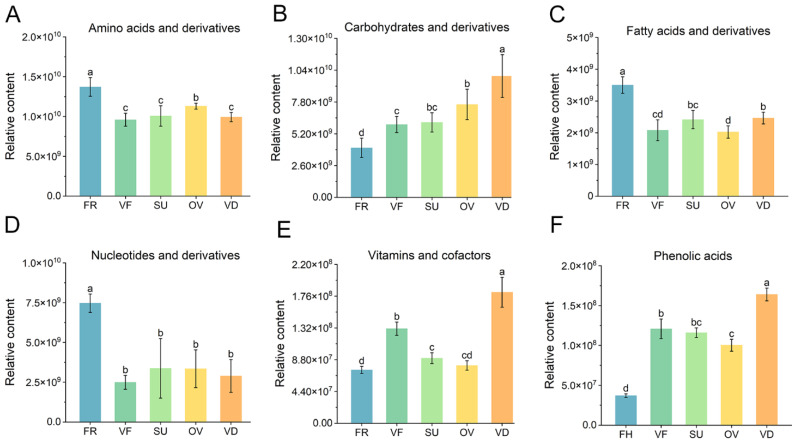
Comparison of differential metabolites between fresh and dried *C. militaris*. Relative total contents of amino acids and derivatives (**A**), carbohydrates and derivatives (**B**), fatty acids and derivatives (**C**), nucleotides and derivatives (**D**), vitamins and cofactors (**E**), and phenolic acids (**F**). Means with different superscript letters are significantly different (*p* < 0.05, ANOVA).

In this study, we identified 41 differential amino acids and their derivatives (AAs), with significant variations in their relative abundances between fresh and dried samples (Table S2). As shown in Figure 4A, the relative levels of total AAs were significantly higher in the fresh and OV groups than in the other three drying methods, suggesting that oven drying better preserved total AAs. Carbohydrates, encompassing both free sugars and polymeric saccharides, serve not only as primary substrates for energy metabolism but also hold significant roles in nutrition and therapeutic applications. A total of 23 carbohydrates and their derivatives were found to be significantly affected by the drying treatments. In summary, all dried samples contained more total carbohydrates and derivatives than the fresh type (Figure 4B). Fatty acids (FAs) not only serve as building blocks of membrane lipids but also display a diversity of physiological functions such as energy supply and regulation of intracellular signaling pathways. It has been shown that drying methods significantly affect the FAs profiles of mushrooms [5]. In our investigation, the fresh *C. militaris* had higher amounts of total differential FAs and their derivatives as compared to the samples treated with different drying methods (Figure 4C), which was in accordance with the previous results from oyster mushroom [23]. The drying process resulted in a decrease in FAs contents of *C. militaris*, which may be explained by the fact that various FAs are catalyzed to form numerous odor-active volatile compounds under the action of some enzymes during drying process such as aldehydes, ketones, and lactones [5].

The nucleotides, nucleosides, and their derivatives present in mushrooms are crucial in modulating various physiological functions in the human body. In this work, we observed significant changes in the levels of 25 nucleotide-related metabolites before and after drying (Table S2). The total content of these differential nucleotides was markedly higher in fresh samples than in dried ones, while no notable differences emerged among the various drying groups (Figure 4D). *C. militaris* contains a variety of water-soluble vitamins and fat-soluble vitamins [1]. As shown in Table S2, a total of 8 vitamins and derivatives exhibited significant changes between fresh and dried samples. The total relative content of these vitamins was higher in dried samples (Figure 4E), indicating that drying treatment may promote the formation of certain vitamins. Phenolic acids (PAs), a key subclass of phenolic compounds present in edible mushrooms, exhibit strong antioxidant activity as well as other health benefits. As illustrated in Figure 4F, the total levels of 7 differential PAs were consistently higher in dried *C. militaris* compared to the fresh samples, regardless of the drying method employed. Notably, vacuum drying produced the highest total PAs content, followed by vacuum freeze drying and sun drying.

### 3.6. Potential Mechanisms of Drying Treatment in C. militaris

**Figure 5:**
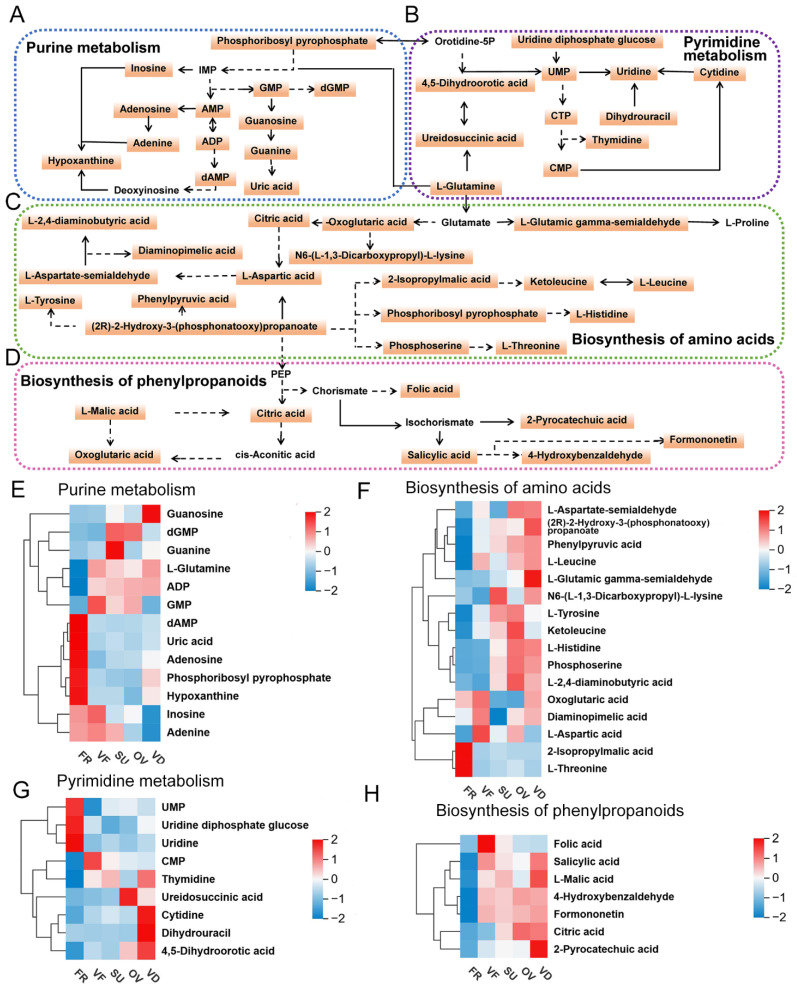
Metabolic pathway network of significantly differential metabolites (SDMs). (**A**) Purine metabolism. (**B**) Pyrimidine metabolism. (**C**) Biosynthesis of amino acids. (**D**) Biosynthesis of phenylpropanoids. Direct connections between metabolites are shown with solid arrows, whereas indirect connections are depicted with dashed arrows. Metabolites highlighted in orange boxes represent SDMs. Heatmap showing the relative abundances of compounds associated with metabolic pathway networks in fresh and dried *C. militaris*. (**E**) Purine metabolism. (**F**) Biosynthesis of amino acids. (**G**) Pyrimidine metabolism. (**H**) Biosynthesis of phenylpropanoids.

To explore the potential mechanisms underlying differential metabolite accumulation during drying treatment, KEGG pathway enrichment analysis was performed based on the identified differential metabolites. The KEGG enrichment results showed that purine metabolism, pyrimidine metabolism, biosynthesis of amino acids, and biosynthesis of phenylpropanoids were the four significantly enriched pathways. Furthermore, these four metabolic pathways are associated with the synthesis of nutrients in *C*. *militaris*. In addition, these pathways were closely associated with the major metabolite classes altered during drying treatment, including nucleotides, amino acids, and phenolic compounds. Based on these results, an integrated metabolic pathway network related to drying-induced metabolic changes in *C. militaris* was constructed (Figure 5A–D).

Phosphoribosyl pyrophosphate is an important precursor involved in both purine and pyrimidine metabolism (Figure 5A,B). In the present study, drying treatment reduced the level of phosphoribosyl pyrophosphate and was accompanied by alterations in several metabolites associated with purine and pyrimidine metabolism (Figure 5E). Specifically, guanosine monophosphate (GMP) and cytidine monophosphate (CMP) contents increased in VF-, SU-, and OV-treated samples, whereas inosine was elevated in VF-treated samples and cytidine and guanosine were increased in VD-treated samples (Figure 5E,G). Previous studies have shown that guanosine, inosine, and cytidine are involved in various physiological functions, particularly in the nervous system [24]. In addition, GMP and CMP are important nonvolatile flavor compounds in edible mushrooms. Among them, GMP contributes to savory and umami taste characteristics and exhibits a stronger umami-enhancing effect than monosodium glutamate [8]. These results suggested that drying treatment may influence both the functional and flavor-related properties of *C. militaris*.

L-Glutamine is not only involved in purine and pyrimidine metabolism but also plays an important role in amino acid biosynthesis (Figure 5A–C). In this study, increased L-glutamine levels after drying were accompanied by elevated accumulation of most differential metabolites associated with amino acid biosynthesis pathways (Figure 5E). Several proteinogenic amino acids, including L-aspartic acid, L-glutamine, L-histidine, L-leucine, and L-tyrosine, showed higher levels in dried samples than in fresh *C. militaris* (Figure 5E,F). Similar increases in free amino acid contents after drying have also been reported in shiitake mushrooms [25]. These findings suggested that drying treatment may contribute to the retention or accumulation of certain amino acids in *C. militaris*.

(2R)-2-Hydroxy-3-(phosphonatooxy) propanoate is a metabolite associated with both amino acid biosynthesis and phenylpropanoid biosynthesis pathways (Figure 5C,D). In dried samples, increased levels of these metabolites were accompanied by elevated accumulation of several differential metabolites involved in phenylpropanoid biosynthesis (Figure 5H). Among these metabolites, L-malic acid, folic acid, 4-hydroxybenzaldehyde, and formononetin have previously been reported to possess antioxidant-related activities [26,27,28,29]. Therefore, the altered phenylpropanoid-related metabolites observed after drying may be associated with the enhanced antioxidant capacity of dried *C. militaris*.

Overall, drying treatment induced substantial alterations in metabolites associated with purine metabolism, pyrimidine metabolism, amino acid biosynthesis, and phenylpropanoid biosynthesis. These metabolic changes may contribute to the differences in flavor characteristics, nutritional composition, and antioxidant-related quality observed among different drying treatments of *C. militaris*.

### 3.7. Network Pharmacology-Based Mechanism Prediction of Antioxidant Activities

**Figure 6:**
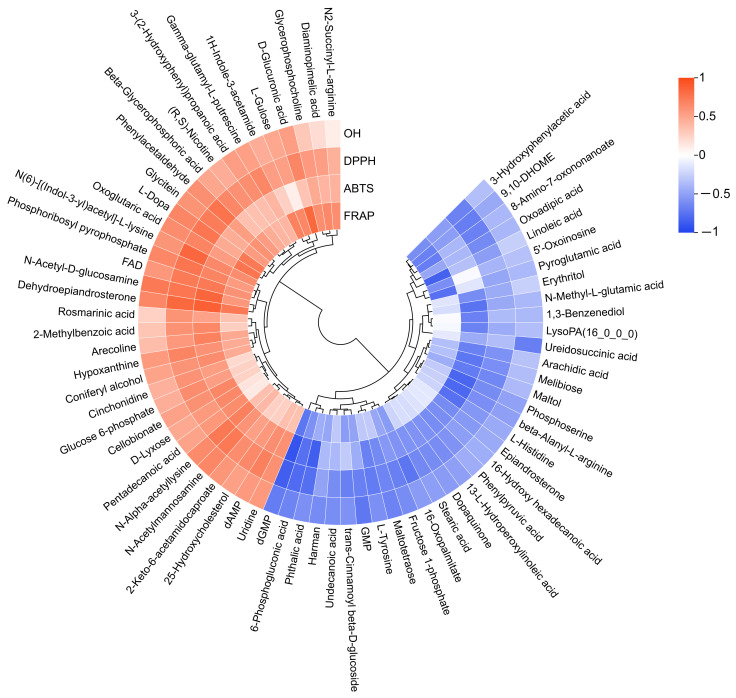
Correlation coefficient heatmap between metabolites and antioxidant activity in *C. militaris*.

**Figure 7:**
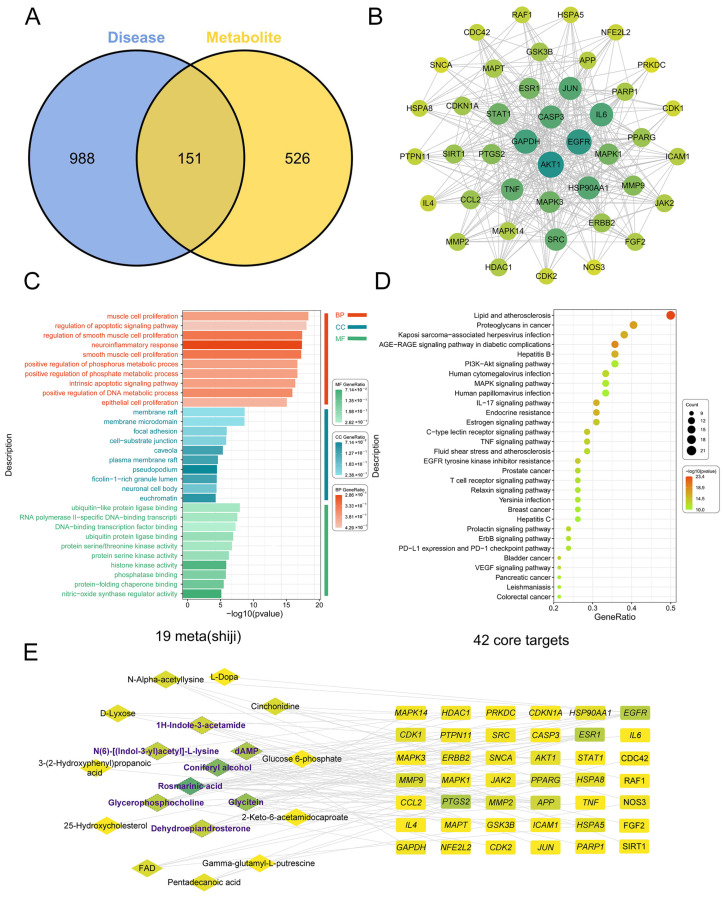
Network pharmacological analysis of antioxidant-related compounds (ARCs) in *C. militaris.* (**A**) Venn diagram of overlapping targets between ARCs and oxidative damage. (**B**) Protein–protein interaction network of 42 core antioxidant targets. (**C**) GO enrichment analysis of the top 30 GO functional terms. (**D**) KEGG pathway enrichment analysis of the top 30 pathways. (**E**) Network analysis of the ARCs and 42 core targets. The diamond and rectangle nodes represent metabolites and targets, respectively. The italic fonts represent targets associated with core metabolites. The color scale represents degree values from low (yellow color) to high (green color). Purple indicates key components contributing to the antioxidant effect in *C. militaris*.

Pearson correlation analysis was conducted to identify metabolites potentially contributing to the antioxidant capacity of *C. militaris*. As shown in Figure 6 and Table S3, a total of 70 metabolites were significantly associated with antioxidant activity (|*r*| > 0.6, *p* < 0.05). Among these, 35 metabolites exhibited positive correlations with antioxidant performance and were therefore defined as antioxidant-related compounds (ARCs). Metabolomics results revealed that the levels of most ARCs were significantly affected by different drying treatments (Figure S4), indicating their potential contribution to the altered antioxidant activity of *C. militaris*. To explore the potential functional associations between ARCs and antioxidant-related targets, network pharmacology analysis was subsequently applied. The chemical structures of the 35 ARCs were retrieved from the PubChem database. After consolidating and removing duplicate records, 677 potential protein targets related to 35 metabolites were obtained from SwissTargetPrediction and TCMSP databases. Meanwhile, 1139 targets associated with oxidative damage were gathered from the GeneCards and OMIM databases. Venn analysis yielded 151 overlapping targets between ARCs and oxidative damage (Figure 7A). These overlapping targets may represent key molecular nodes linking drying-induced metabolite variations to antioxidant activity of *C. militaris*.

Subsequently, the 151 shared targets were imported into STRING for protein–protein interactions (PPI) analysis to highlight the core targets within modules. Using Cytoscape, a high-confidence PPI network (interaction score > 0.7) was constructed based on 143 intersecting targets. The CytoNCA plugin in Cytoscape was used to identify 42 core targets related to oxidative damage in *C. militaris* based on median values of multiple topological parameters, including betweenness, closeness, degree, eigenvector, local average connectivity (LAC), and network scores (Figure 7B). Several of these hub targets have been documented to participate in oxidative stress regulation, highlighting their potential roles in antioxidant mechanisms of *C. militaris*. For instance, AKT1 functions as a serine/threonine kinase and is implicated in diverse pathways, including cell proliferation, metabolism, and angiogenesis. Previous study reported that olive oils exerted antioxidant activity by inhibiting the AKT1 protein [30]. Interleukins-6 (IL6) and tumour necrotic factor (TNF) are two pro-inflammatory cytokines that can stimulate nicotinamide adenine dinucleotide phosphate (NADPH) oxidases to generate reactive oxygen species (ROS), thus exacerbating oxidative injury [31]. Epidermal growth factor receptor (EGFR), a type of tyrosine kinase receptor, has been shown to promote low-density lipoprotein oxidation and accelerate atherosclerosis progression [32]. Furthermore, cyclooxygenase-2 (COX-2), also referred to as prostaglandin-endoperoxide synthase 2 (PTGS2), plays a crucial role in the biosynthesis of prostaglandins and can generate reactive oxygen species, thereby worsening oxidative stress [33]. Network pharmacology analysis predicted that *C. militaris* metabolites may be associated with antioxidant-related pathways through potential interactions with targets such as AKT1, IL6, TNF, EGFR, and PTGS2.

GO functional annotation and KEGG enrichment analyses were carried out to gain insight into the biological functions of the 42 core targets. The top 10 enriched GO terms in biological process (BP), cellular component (CC), and molecular function (MF) were shown in Figure 7C. For BP, the targets were mainly involved in regulation of apoptotic signaling pathway, epithelial cell proliferation, and muscle cell proliferation (Figure 7C). In the CC category, the targets were primarily associated with membrane raft, membrane microdomain, and focal adhesion (Figure 7C). In terms of molecular function, the targets were primarily associated with DNA-binding transcription factor binding, RNA polymerase II-specific DNA-binding transcription factor binding, and ubiquitin-like protein ligase binding (Figure 7C). KEGG enrichment analysis further showed that the 42 core targets were significantly enriched in 165 pathways. The bubble plot of the top 30 enriched pathways suggested that the identified metabolites may be associated with several antioxidant-related pathways, including lipid and atherosclerosis, AGE–RAGE signaling pathway in diabetic complications, proteoglycans in cancer, and Kaposi sarcoma-associated herpesvirus infection (Figure 7D).

To characterize the interactions between ARCs and core protein targets, a metabolite–target network was constructed (Figure 7E). Eight key components contributing to the antioxidant effect in *C. militaris* were further confirmed based on the medians of topological parameters, including rosmarinic acid, coniferyl alcohol, glycitein, deoxyadenosine monophosphate (dAMP), glycerophosphocholine, N(6)-[(indol-3-yl)acetyl]-L-lysine, dehydroepiandrosterone, and 1H-indole-3-acetamide. These key compounds interacted with 37 core targets (Figure 7E), suggesting their involvement in multiple antioxidant-related processes and contribution to the antioxidant capacity of *C. militaris*. Notably, several core metabolites have been previously reported to possess strong antioxidant or ROS scavenging activities, supporting the reliability of the network pharmacology predictions. For instance, glycitein is a soy isoflavone with a unique 6-methoxy substitution and exhibits significant antioxidant activity by activating the Nrf2-ARE signaling pathway [34]. Rosmarinic acid (RA) demonstrates anti-inflammatory and antioxidant effects by regulating insulin/IGF signaling and MAPK pathways, thereby enhancing antioxidant enzyme expression in *Caenorhabditis elegans* [35]. Coniferyl alcohol is considered as a potent antioxidant reported to outperform the standard phenolic antioxidant butylated hydroxytoluene [36]. Dehydroepiandrosterone has been shown to exert antioxidative effects by reducing lipid peroxidation, preventing membrane fluidity loss during aging, and protecting neuronal mitochondria against calcium overload [37].

Importantly, four core targets consisting of PTGS2, ESR1, amyloid precursor protein (APP), and EGFR displayed higher values in degree, betweenness, and closeness centrality at the same time (Figure 7E), indicating their indispensable roles in oxidative damage related signaling. In fact, several phenolic compounds such as apigenin and rosmarinic acid exhibit antioxidant effects by inhibiting the activity of PTGS2 [38]. Selective activation of estrogen receptor α (ESR1) reduces oxidative injury in vascular smooth muscle cells under hyperglycemia [39]. Another study reported that red wine polyphenols enhance endothelial function through ESR1 activation by increasing nitric oxide and reducing superoxide anions levels [40]. Tea polyphenols have been reported to inhibit EGFR activation by blocking its binding to EGF, thereby reducing EGFR autophosphorylation and subsequent oxidative stress [41]. In addition, amyloid precursor protein (APP) serves as the precursor of amyloid-β (Aβ), which promotes excessive free-radical formation and consequent oxidative damage in brain neurons during the pathogenesis of Alzheimer’s disease [42]. Furthermore, the previous metabolomics results indicated that different drying methods exhibited heterogeneous effects on the retention of these core metabolites. Rosmarinic acid, glycitein, dAMP, and N(6)-[(indol-3-yl)acetyl]-L-lysine were significantly decreased after drying, whereas coniferyl alcohol remained relatively stable (Figure S4). Overall, vacuum drying and vacuum freeze-drying preserved higher levels of several core ARCs, such as rosmarinic acid, glycerophosphocholine, and dehydroepiandrosterone. Consistently, antioxidant assays also supported that samples processed by freeze-drying and vacuum drying exhibited stronger radical scavenging capacities and higher FRAP values compared with oven-dried and sun-dried samples (Figure 1F–I). Taken together, these findings suggest that the superior antioxidant efficacy observed in the freeze-dried and vacuum-dried *C. militaris* may derive from the enhanced retention of critical metabolites that contribute to mitigating oxidative stress.

### 3.8. Limitations of the Study

In this study, metabolite identification was primarily based on MS/MS spectral matching with self-built and public databases, additional confirmation using authentic standards together with targeted quantitative analysis would further improve the accuracy and reliability of metabolite annotation. Although network pharmacology analysis provided preliminary insights into the potential antioxidant-related functions of key metabolites, these predictions were mainly derived from computational analysis and correlation results, and therefore require further validation through in vitro and in vivo experiments. Because different drying methods involve multiple processing factors simultaneously, future studies under more controlled drying conditions are required to further distinguish the individual effects of specific processing parameters on metabolite stability and quality changes. In addition, the *C. militaris* samples analyzed in this study were obtained from a single geographic origin, and future investigations using samples from multiple origins would help to further evaluate the general applicability of the present findings.

## 4. Conclusions

In this study, the influence of different drying techniques, namely vacuum freeze drying (VF), oven drying (OV), sun drying (SU), and vacuum drying (VD) on the bioactive compounds, metabolic characteristics, and antioxidant properties of *C. militaris* were explored. The findings showed that the drying process caused a notable reduction in the levels of total phenolics, total carotenoids, and N^6^-(2-hydroxyethyl)-adenosine. VF and OV exhibited better retention for these above compounds than SU and VD. VD and VF samples presented the highest levels of total flavonoids and cordycepin, respectively. In addition, VF- and VD-treated samples exhibited stronger antioxidant capacities than SU- and OV-treated samples in DPPH•, ABTS^•+^, •OH, and FRAP assays. Metabolomics analysis identified 431 metabolites, including 193 differential metabolites between fresh and dried *C. militaris*. Drying tended to significantly decrease the relative contents of nucleotides and nucleosides, fatty acids, and their derivatives. In contrast, drying resulted in an obvious increase in the relative levels of carbohydrates and phenolic acids. KEGG enrichment analysis further revealed significant alterations in purine metabolism, pyrimidine metabolism, amino acid biosynthesis, and phenylpropanoid biosynthesis. Additionally, network pharmacology analysis suggested that 8 key compounds such as glycitein and rosmarinic acid likely responsible for antioxidant effect of *C. militaris*, primarily through their interaction with core targets such as PTGS2, ESR1, and APP. These findings provide useful insights into the effects of different drying techniques on the quality characteristics of *C. militaris* and may support the selection of suitable drying methods for different processing purposes.

## Data Availability

The original contributions presented in the study are included in the article/Supplementary Materials. Further inquiries can be directed to the corresponding authors.

## Supplementary Materials

The following supporting information can be downloaded at: https://www.mdpi.com/article/10.3390/foods15122061/s1, Figure S1: Principle component analysis of five injections of the quality control (QC) samples in positive (A) and negative (C) ion modes. Relative standard deviation (RSD) distribution for all ion features in positive (B) and negative (D) ion modes; Figure S2: Score plots of orthogonal partial least squares discriminant analysis (OPLS-DA) for the following comparison groups: between VF and FR from positive (A) and negative (B) ion modes; between OV and FR from positive (C) and negative (D) ion modes; between SU and FR from positive (E) and negative (F) ion modes; between VD and FR from positive (G) and negative (H) ion modes; Figure S3: The permutation test for OPLS-DA models of the four comparisons: between VF and FR groups in positive (A) and negative (B) ion and modes, between OV and FR groups in positive (C) and negative (D) ion and modes, between SU and FR groups in positive (E) and negative (F) ion and modes, between VD and FR groups in positive (G) and negative (H) ion and modes; Figure S4: Heatmap of relative contents of antioxidant-related compounds in fresh and dried *C. militaris*; Table S1: List and characteristics of the metabolites identified and quantified in fresh and dried *Cordyceps militaris*; Table S2: List of significantly differential metabolites (SDMs) between fresh and dried *C. militaris* under different drying methods; Table S3: Pearson correlation coefficient between the metabolites and antioxidant capacity of *C. militaris*.
