# Supplementary material for: Drying-Induced Changes in Metabolite Profiles and Antioxidant Activity of Cordyceps militaris: Insights from Integrated Metabolomics and Network Pharmacology
Source: Foods. 2026 Jun 7;15(12):2061. doi: 10.3390/foods15122061 (PMC13298478; doi:10.3390/foods15122061)
Supplement: Supplementary file 1 [file foods-15-02061-s001.zip › Supplementary FigureS1-4.pdf]

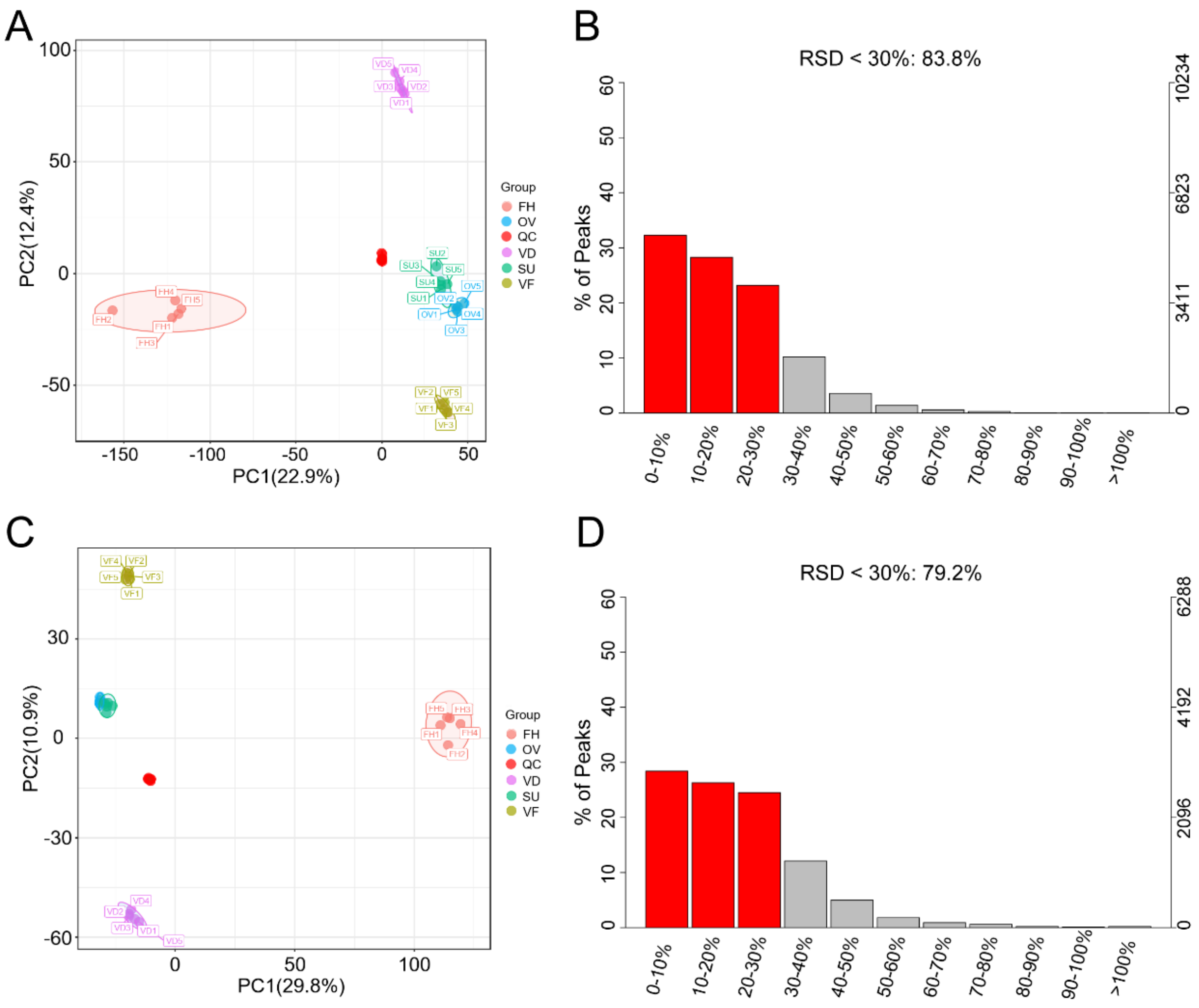

Figure S1. Principle component analysis of five injections of the quality control (QC) samples in positive (A) and negative (C) ion modes. Relative standard deviation (RSD) distribution for all ion features in positive (B) and negative (D) ion modes.

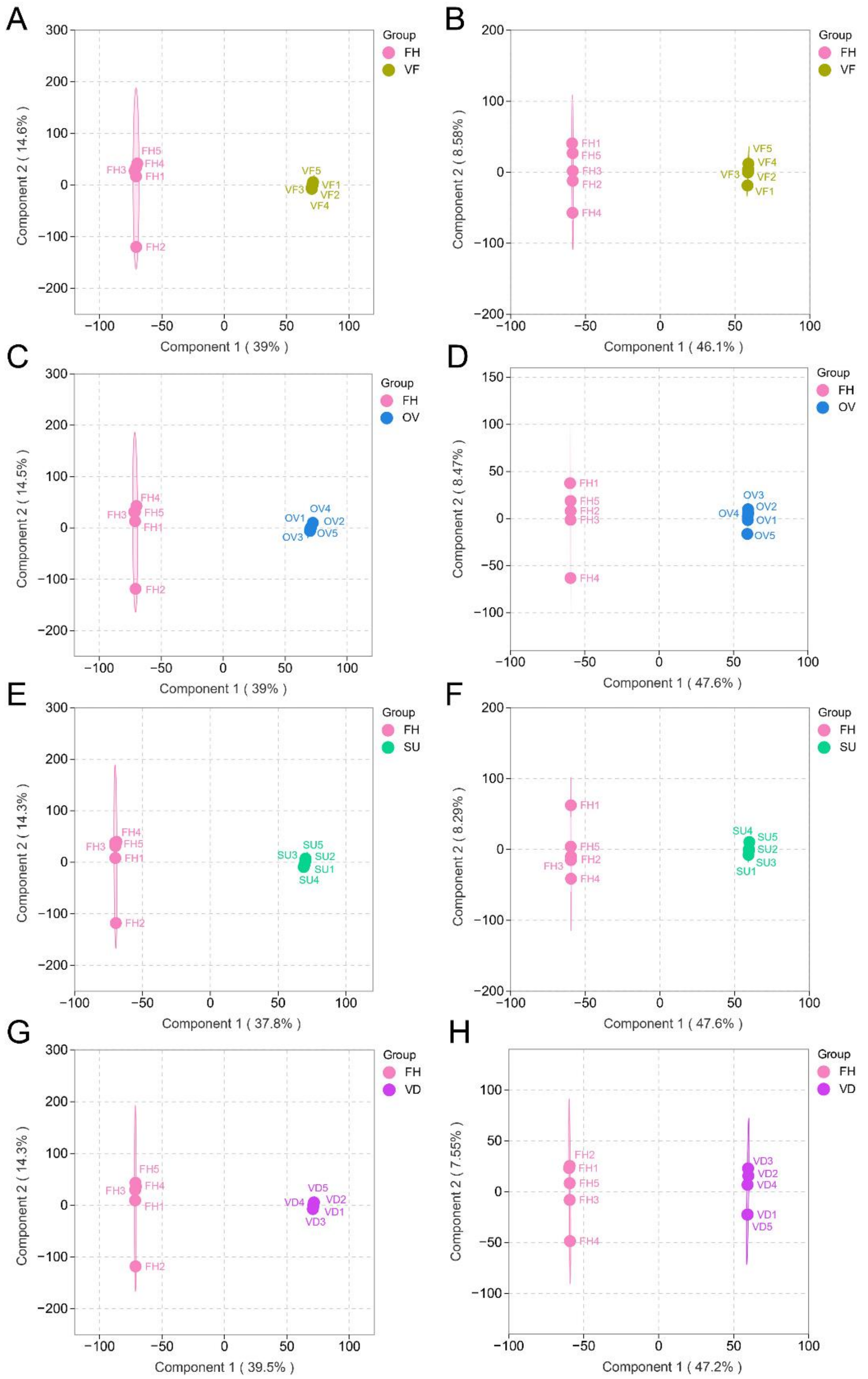

Figure S2. Score plots of orthogonal partial least squares discriminant analysis (OPLS-DA) for the following comparison groups: between VF and FH from positive (A) and negative (B) ion modes; between OV and FH from positive (C) and negative (D) ion modes; between SU and FH from positive (E) and negative (F) ion modes; between VD and FH from positive (G) and negative (H) ion modes.

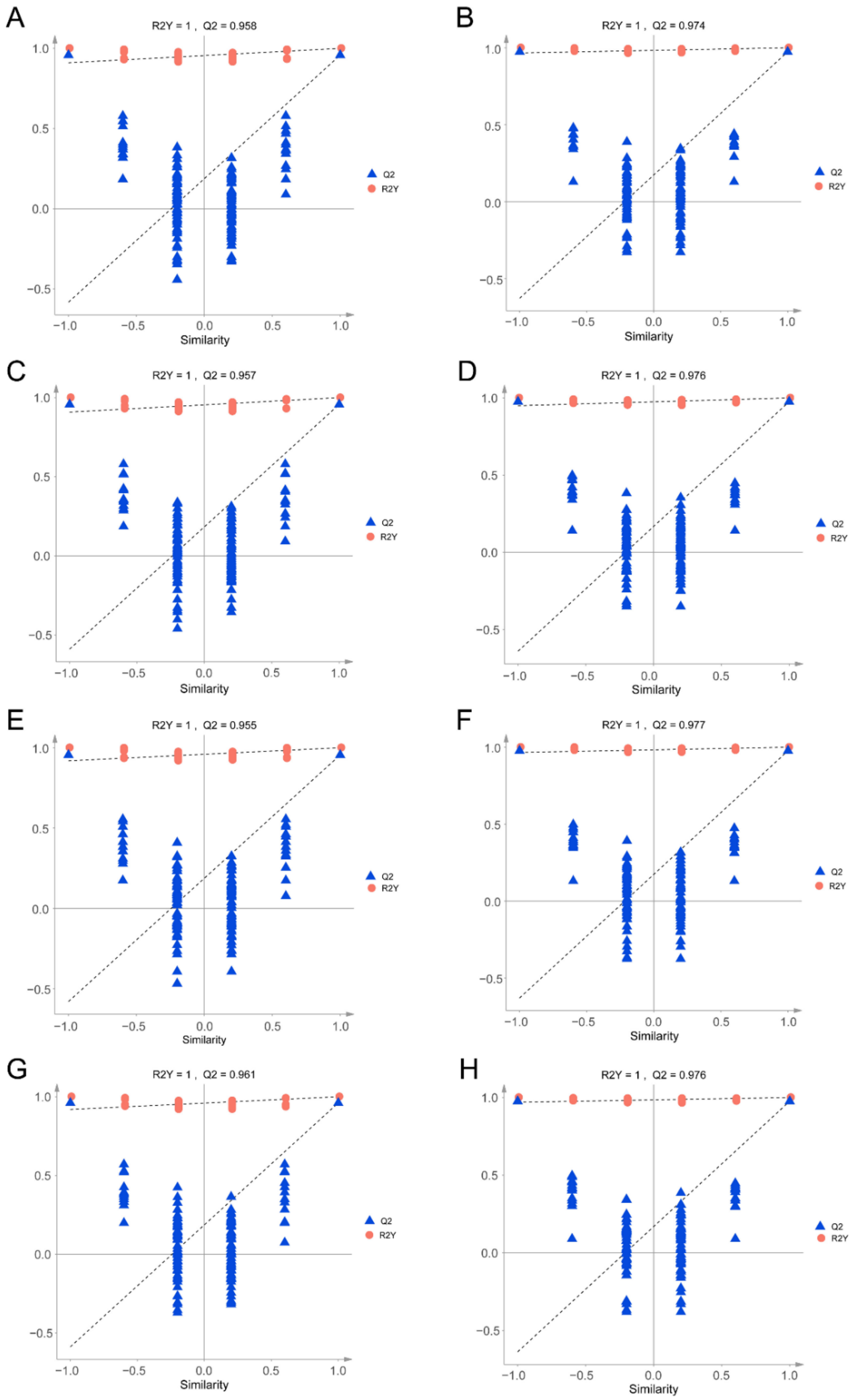

Figure S3. The permutation test for OPLS-DA models of the four comparisons: between VF and FH groups in positive (A) and negative (B) ion and modes, between OV and FH groups in positive (C) and negative (D) ion and modes, between SU and FH groups in positive (E) and negative (F) ion and modes, between VD and FH groups in positive (G) and negative (H) ion and modes.

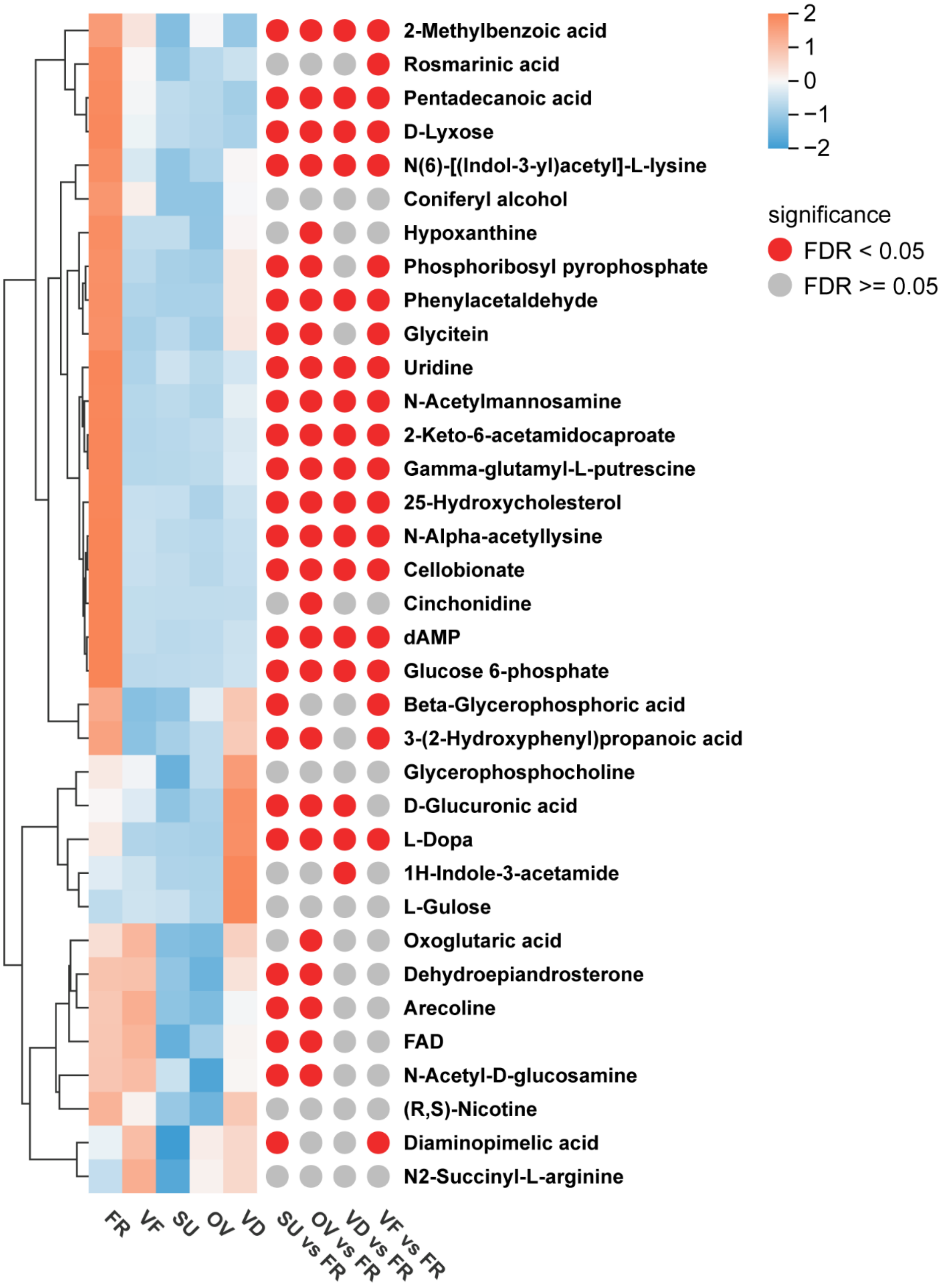

Figure S4. Heatmap of relative contents of antioxidant-related compounds in fresh and dried *C. militaris*.
